# Genetic Risk Profiling Reveals Altered Glycosyltransferase Expression as a Predictor for Patient Outcome in Neuroblastoma

**DOI:** 10.3390/jcm14020527

**Published:** 2025-01-15

**Authors:** Isabelle Ariane Bley, Stefan Behrens, Michael Spohn, Ingo Müller, Benjamin Schattling

**Affiliations:** 1 Research Institute Children’s Cancer Center Hamburg, 20251 Hamburg, Germany; 2 Division of Pediatric Stem Cell Transplantation and Immunology, Clinic of Pediatric Hematology and Oncology, University Medical Center Hamburg-Eppendorf, 20246 Hamburg, Germany; 3 Bioinformatics Core, University Medical Center Hamburg-Eppendorf, 20246 Hamburg, Germany

**Keywords:** neuroblastoma, immune evasion, MYCN, glycosylation

## Abstract

**Background/Objectives**: Neuroblastoma is a highly aggressive pediatric cancer that arises from immature nerve cells and exhibits a broad spectrum of clinical presentations. While low- and intermediate-risk neuroblastomas often have favorable outcomes, high-risk neuroblastomas are associated with poor prognosis and significant treatment challenges. The complex genetic networks driving these high-risk cases remain poorly understood. This study aims to investigate differences in gene expression patterns that may contribute to disease outcomes. **Methods**: We employed an in silico approach to analyze a cohort of 493 neuroblastoma tumor samples that underwent mRNA sequencing (GSE49711). This dataset was reanalyzed in depth with a non-hypothesis-driven approach to identify the expression patterns and regulatory mechanisms associated with a poor prognosis. **Results**: By exploring global gene expression and the integration of clinical parameters, we stratified the samples into two groups with highly distinct gene expression profiles. MYCN amplification emerged as a major driver not only of poor prognosis but also of specific gene regulatory patterns. Notably, tumors with MYCN amplification exhibited the strong regulation of immune response genes and less immune infiltration, suggesting potential immune evasion. However, while we observed only minor changes in immune checkpoint expression, there was a strong modulation of glycosyltransferase genes in MYCN-amplified tumors. Using this information, we were able to construct a risk profile based on 12 glycosylation-related genes, which correlates with the survival outcomes of neuroblastoma patients. **Conclusions**: This study highlights the role of MYCN amplification in driving a poor prognosis in neuroblastoma through the regulation of immune response and glycosylation-related genes. Based on this finding, we developed a genetic risk profile that correlates with survival outcomes in neuroblastoma patients.

## 1. Introduction

Neuroblastoma (NB) is a leading cause of cancer-related mortality in young children, arising from neural crest cells of the sympathetic nervous system. Despite its prevalence, NB remains relatively unique among pediatric cancers due to its highly variable clinical course: while some cases spontaneously regress or respond well to therapy, others are resistant to treatment and associated with poor outcomes [1,2,3]. Classification into risk groups is based on factors such as the patient’s age, tumor stage, histological features, and MYCN oncogene amplification, a key driver of aggressive disease behavior [4]. Furthermore, different gene expression-based classifiers have been developed to define the risk profiles in neuroblastoma patients [5,6,7,8,9]. However, the molecular mechanisms that underpin this prognostic divide, particularly in high-risk NB, remain poorly defined.

MYCN amplification is one of the most significant molecular alterations in NB and is strongly associated with aggressive disease and poor prognosis. MYCN is a transcription factor that regulates various cellular processes, including cell cycle progression, apoptosis, and differentiation [10,11]. In NB, MYCN amplification leads to dysregulated cell growth and resistance to chemotherapy, contributing to the high-risk profile of affected tumors [12]. While MYCN-driven tumorigenesis has been well studied, the mechanisms by which MYCN amplification confers a survival advantage to the tumor cells, especially in the context of immune evasion, remain less understood.

Immune evasion is a critical factor in the progression of many cancers, including NB [13]. Tumors can evade detection and destruction by the immune system through various strategies, such as the regulation of immune checkpoint molecules, which can inhibit immune cell infiltration, and the modulation of immune response pathways [14]. In NB, evidence suggests that immune evasion may play a key role in the failure of current therapies and the poor outcomes observed in high-risk patients [15].

Glycosylation is a fundamental post-translational modification that plays a crucial role in numerous cellular processes, including protein folding, signaling, and immune recognition [16]. It involves the attachment of sugar moieties to proteins and lipids, altering their structure and function. Glycosyltransferases are enzymes that catalyze the transfer of specific sugar residues to acceptor molecules, and their activity is tightly regulated in both normal and pathological conditions. In cancer, aberrant glycosylation patterns have been implicated in tumor progression, metastasis, and immune evasion [17]. Alterations in glycosyltransferase gene expression can lead to the generation of altered glycan structures that influence tumor–host interactions, immune cell recognition, and the immune response. In neuroblastoma, some evidence suggests that changes in glycosylation, mediated by dysregulated glycosyltransferase activity, may contribute to immune evasion and resistance to therapy, highlighting the potential of glycosylation pathways as both prognostic markers and therapeutic targets [18].

In this study, we performed a large-scale in silico analysis of neuroblastoma samples to identify the gene expression patterns associated with a poor prognosis. Notably, MYCN amplification emerged as a significant predictor of survival outcomes and a key driver of differential gene expression in the tumor samples. This led us to investigate the genetic networks altered by MYCN amplification. Interestingly, we observed the pronounced deregulation of immune response pathways and decreased immune cell infiltration, although immune checkpoint regulation was only mildly affected in these high-risk samples with a poor prognosis. In our search for a potential underlying mechanism, we identified a strong dysregulation of glycosyltransferase gene expression. This discovery allowed us to establish a 12-gene risk signature that correlates with survival outcomes in neuroblastoma, providing valuable insights into potential prognostic biomarkers and therapeutic targets for high-risk neuroblastoma.

## 2. Materials and Methods

### 2.1. Data Acquisition and Processing

Gene expression data (“GSE49711_SEQC_NB_TUC_G_log2”) and corresponding clinical information for the neuroblastoma cohort GSE49711 were obtained from the publicly available Gene Expression Omnibus (GEO) database [19]. Patients with an unavailable MYCN status were excluded, resulting in a cohort of 493 samples, as detailed in Table 1. The gene expression data were filtered to retain only protein-coding genes using a reference list from the NCBI database (https://www.ncbi.nlm.nih.gov/datasets/gene/taxon/9606/?gene_type=protein-coding (accessed on 16 December 2024)). Genes with low expression (mean expression < 0.1) and low variance (variance < 0.1) across samples were further excluded to minimize background noise and concentrate on genes with meaningful biological variability. Gene expression data and corresponding clinical information for the validation data set GSE16476 [20] were obtained from the R2 platform (R2: Genomics Analysis and Visualization Platform, http://r2.amc.nl (accessed on 16 December 2024)). To normalize the gene expression data of the validation data set, a log2 transformation was applied.

### 2.2. Identification of Differentially Expressed Genes (DEGs) Between MYCN-A and MYCN-NA Samples

Sample similarity was visualized using t-Distributed Stochastic Neighbor Embedding (t-SNE) via the “Rtsne” package, which reduces high-dimensional data into a lower-dimensional space while preserving relative data point similarities. Differential gene expression analysis comparing MYCN-amplified (MYCN-A) versus MYCN-non-amplified (MYCN-NA) samples was conducted with the “limma” package. DEGs were defined via logFC > 0.5 and logFC < −0.5 and an adjusted *p*-value < 0.01, applying the Benjamini–Hochberg correction for controlling the false discovery rate.

### 2.3. Functional Enrichment Analysis and Gene Set Enrichment Analysis

Gene Ontology (GO) term enrichment analysis was performed with the “clusterProfiler” package to identify significantly enriched biological processes (BP) for the top 250 upregulated and 250 downregulated genes based on fold-change values. A *p*-value and adjusted *p*-value cutoff of 0.05 was used, with multiple testing correction performed via the Benjamini–Hochberg method. Redundant terms were minimized using the “simplify()” function in “clusterProfiler” (cutoff = 0.7) to focus on the most representative biological processes for interpretability, and representative terms were selected based on adjusted *p*-values. To visualize the biological processes enriched in MYCN-A neuroblastoma compared to MYCN-NA neuroblastoma, enrichment maps were generated using the “enrichplot” package and subsequently refined for clarity. To determine whether biological states or processes represented by Hallmark gene sets were enriched in MYCN-A samples, we conducted Gene Set Enrichment Analysis (GSEA), a computational method used to identify the statistically significant enrichment of predefined gene sets in a ranked list of genes. The analysis was performed on a ranked list of DEGs using the “clusterProfiler” package, using Hallmark gene sets (“h.all.v2024.1.Hs.symbols.gmt”) from MSigDB as references [21]. Immune-related Hallmark pathways were further selected and highlighted in a gene concept network plot using “cnetplot()” from “enrichplot”.

### 2.4. Immune Profiling and ImmuneScore-Dependent Survival Analysis

The ESTIMATE (Estimate of STromal and Immune Cells in Malignant Tumor Tissues from Expression Data) algorithm [22], a computational method that infers the proportions of immune and stromal cells in tumor samples based on gene expression profiles, was applied to explore the immune microenvironment differences associated with the MYCN amplification status. To evaluate the impact of immune infiltration on overall survival, the optimal cutoff for the immune score was determined using the “surv_cutpoint()” function, dividing the cohort into ImmuneScore^High^ and ImmuneScore^Low^ groups. Kaplan–Meier survival analysis was performed using the “survival” and “survminer” package. The alluvial plot created with the “ggalluvial” extension of “ggplot2” visualized the distribution and relationship between the MYCN amplification status and immune score groups. The “MCPcounter” package, a computational tool designed to quantify the abundance of specific immune and stromal cell populations from transcriptomic data, was utilized to estimate the cell infiltration levels in the samples.

### 2.5. Immune Checkpoint and Cell Surface Protein Analysis

The expression of already known immune checkpoints described in the literature was explored in the dataset. A comprehensive reference list of cell surface proteins was generated by merging gene sets obtained from the HUGO Gene Nomenclature Committee (HGNC) (CD molecules) and the Human Protein Atlas (Plasma Membrane) (Supplementary Table S1). Differentially expressed genes (DEGs) in MYCN-A samples were then intersected with this reference list. The expression of the top 50 upregulated and downregulated cell surface genes, ranked by fold change, was visualized using a heatmap. The functional enrichment analysis of molecular functions (MF) was performed using the top 250 upregulated and downregulated cell-surface-related genes, employing the “clusterProfiler” package. To reduce redundancy, the GO terms were further simplified using the “simplify()” function based on semantic similarity. Gene concept network plots illustrating the relationships between genes and their associated GO terms were generated using the “cnetplot()” function from the “enrichplot” package.

### 2.6. Construction and Validation of a Glycosyltransferase-Related Risk Signature

Alterations in gene expression related to glycosylation were evaluated using the gene set “GOBP_Glycosylation” from the GO Term GO:0070085. Glycosyltransferase gene lists were obtained from the HUGO Gene Nomenclature Committee (HGNC) database (https://www.genenames.org/data/genegroup/#!/group/424) (accessed on 16 December 2024). To identify glycosyltransferase genes within DEGs associated with MYCN status, overlaps between the glycosyltransferase gene set and the DEGs were determined. The prognostic value of each identified glycosyltransferase gene was assessed through univariate Cox proportional hazards regression using overall survival data and the “survival” R package. Genes with *p*-values < 0.05 were considered statistically significant, and multiple testing correction was applied to control for false discovery rates. To further refine the prognostic gene signature, Least Absolute Shrinkage and Selection Operator (LASSO)-penalized Cox regression was performed using the “glmnet” package. The optimal lambda value (lambda.1se) was determined through cross-validation to balance the model complexity and predictive accuracy. Hereby, lambda.1se was chosen over lambda.min as it provides a more streamlined model with fewer variables, reducing the risk of overfitting while maintaining a robust predictive performance. Genes with non-zero coefficients were retained for further analysis, as they were identified as having a significant association with patient outcomes. For each patient, a glycosyltransferase-related risk score was calculated using the expression values of LASSO-selected genes and their corresponding coefficients derived from the LASSO Cox model. The glycosyltransferase-based risk score for each patient was calculated as follows:

GT−RiskScore=Σ(βi×xi)

where βi is the LASSO-derived coefficient for gene *i* and xi is the expression level of gene *i* for that patient. Patients were stratified into high-risk (GT-RiskScore^High^) and low-risk (GT-RiskScore^Low^) groups based on the median risk score. Kaplan-Meier survival analysis was performed using the “survival” and “survminer” R packages to assess the prognostic value of the risk signature in relation to the overall survival probability.

### 2.7. Time-Dependent Receiver Operating Characteristic (ROC) Curve Analysis

Time-dependent Receiver Operating Characteristic (ROC) curve analysis was employed to evaluate the sensitivity and specificity of the glycosyltransferase-based risk signature in predicting 3-year and 5-year survival outcomes. The ROC curve illustrates the balance between sensitivity (true positive rate) and specificity (false positive rate), with the area under the curve (AUC) serving as an indicator of the model’s overall predictive performance. To further assess the prognostic value of the glycosyltransferase-based risk score, separate ROC analyses were performed for clinical factors such as the MYCN status, clinical high risk categorization, and INSS stage, allowing a direct comparison of their predictive performance.

### 2.8. Correlation Analysis Between Glycosyltransferase-Based Risk Score and Clinical Characteristics

The relationship between the GT-RiskScore and various clinical characteristics, including MYCN amplification status, age at diagnosis, clinical high risk status and INSS stage, was assessed using statistical tests and visualizations, including alluvial and violin plots from the packages “ggalluvial” and “ggplot2”.

### 2.9. Independent Prognostic Analysis

Univariate and multivariate Cox regression analyses were conducted to assess the association between the GT-RiskScore, clinical characteristics and overall survival using the “survival” package. Hazard ratios (HRs), 95% confidence intervals (CIs), and *p*-values were calculated, with false discovery rate (FDR) correction applied to univariate results. The “survminer” and “forestplot” packages were used to generate a forest plot, providing a visual representation of HRs and CIs for both univariate and multivariate analyses, showing the individual and combined effects of the variables.

### 2.10. Statistical Analysis

Statistical analysis was performed through R software (version 4.4.1). Survival curves were generated using the Kaplan–Meier method and differences between survival groups were assessed using the log-rank test. The Wilcoxon rank-sum was used to compare continuous variables between two groups. Bonferroni correction was applied to adjust for multiple comparisons. The statistical significance for all tests was defined as a two-sided *p* < 0.05 unless otherwise noted.

## 3. Results

**Figure 1:**
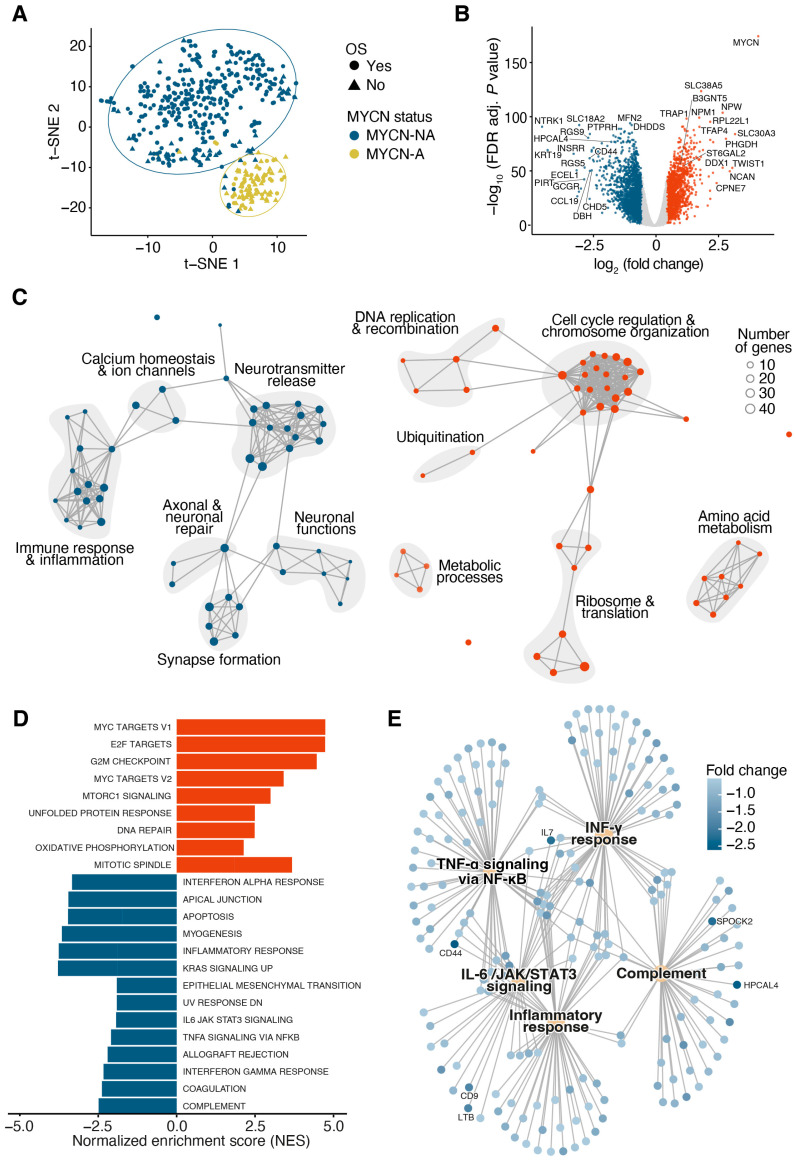
MYCN amplification in neuroblastoma leads to massive gene regulation and changes in the immune response. (**A**) t-SNE plot of 493 neuroblastoma samples from the GSE49711 cohort showing overall survival and MYCN status. Ellipses show 95% confidence intervals. (**B**) Volcano plot of differentially expressed genes (DEGs) between MYCN-A and MYCN-NA samples. Genes are classified as upregulated (logFC > 0.5, adj. *p*-value < 0.01, shown in red), downregulated (logFC < −0.5, adj. *p*-value < 0.01, shown in blue) or not significant (shown in grey). Highly significant or highly regulated genes are labelled. (**C**) Enrichment map of the top 50 most significantly regulated GO biological process terms among the 500 most regulated genes (250 upregulated, red; 250 downregulated, blue) between MYCN-A and MYCN-NA samples. Node size, gene set size; grey lines, gene set overlap. (**D**) Two-sided bar plot showing the most significantly upregulated (red) and downregulated (blue) Hallmark pathways identified by GSEA in MYCN-A compared to MYCN-NA samples. NES indicates the magnitude of pathway enrichment. (**E**) Gene-pathway network of immune-related Hallmark pathways identified through GSEA in MYCN-A compared to MYCN-NA samples. Blue nodes, genes; yellow nodes, Hallmark pathways. Genes with a fold change < −2 are labelled.

Neuroblastoma samples from a large cohort (GSE49711, *n* = 493) were analyzed using an in silico approach to explore the gene expression patterns associated with disease outcomes. Among the cohort, 35.5% were high-risk patients, and 18.7% had MYCN-amplified neuroblastoma. The patient distribution by INSS stage included the following: stage 1 (24.3%), stage 2 (15.8%), stage 3 (12.6%), stage 4 (36.7%), and stage 4S (10.5%). In total, 78.9% of patients had a favorable prognosis, while 21.1% experienced poor outcomes (Table 1). To visualize the distribution of samples based on gene expression, t-SNE analysis was performed and annotated with clinical parameters, including overall survival and the INSS stage (Supplementary Figure S1A), high-risk status (Supplementary Figure S1B), and MYCN status (Figure 1A). This analysis revealed that MYCN-amplified (MYCN-A) samples formed a distinct cluster from MYCN-non-amplified (MYCN-NA) samples, underscoring MYCN as a key driver of differential gene expression. Notably, MYCN-A samples were associated with a higher incidence of poor outcomes, indicating a correlation between MYCN amplification and an unfavorable prognosis. Kaplan–Meier survival analysis further confirmed the significantly worse overall survival for patients with MYCN-A neuroblastoma (Supplementary Figure S1C). The observed separation in the t-SNE plots and survival differences underscores a link between MYCN amplification, gene expression changes, and clinical outcomes.

To further investigate the biological mechanisms behind these differential clinical outcomes, we conducted differential expression analysis between MYCN-A and MYCN-NA samples. This analysis identified 3564 differentially expressed genes (DEGs), with 1346 upregulated and 2218 downregulated, visualized in a volcano plot (Figure 1B). Gene Ontology (GO) analysis of the top 250 upregulated and downregulated genes indicated that upregulated DEGs are primarily associated with cell cycle regulation, chromosome organization, DNA replication and recombination, while downregulated DEGs are involved in synapse formation, neurotransmitter release, and surprisingly in immune response and inflammation (Figure 1C). To further investigate these findings, we conducted an additional gene set enrichment analysis (GSEA) of Hallmark pathways. Here, we could also identify the upregulated pathways connected to cell cycle regulation and DNA repair, as well as the downregulated pathways associated with immune responses, inflammation, and apoptosis regulation (Figure 1D). While both unbiased analyses revealed immune regulatory mechanisms, we further focused on a gene concept network specifically for immune-related Hallmark pathways. This analysis indicated the reduced expression of immune-related genes, such as IL7, CD44, CD9, LTB, SPOCK2, and HPCAL4, which contribute to TNF-α signaling, IL-6/JAK/STAT3 signaling, and complement activation (Figure 1E). These findings suggest that MYCN amplification in neuroblastoma alters the immune response, potentially influencing patient outcomes.

**Figure 2:**
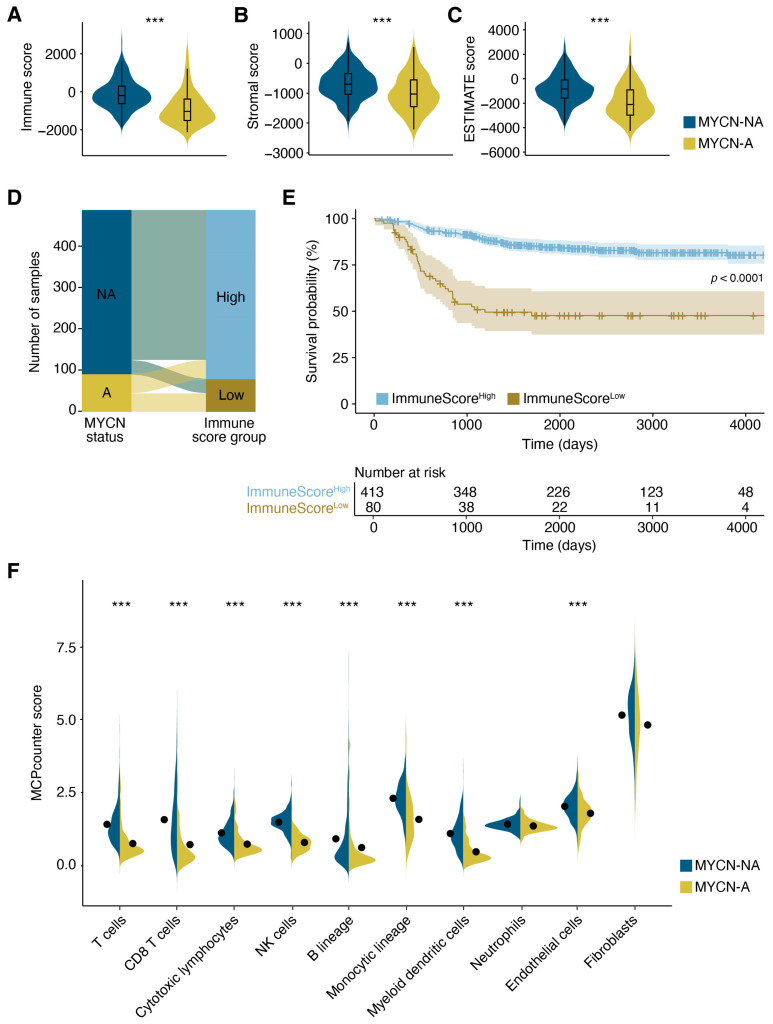
MYCN amplification reduces immune cell infiltration and is associated with poor prognosis in neuroblastoma. ESTIMATE analysis comparing immune cell scores (**A**), stromal scores (**B**) and ESTIMATE scores (**C**) between MYCN-A and MYCN-NA samples. (**D**) Alluvial plot showing dependency between MYCN status and immune scores. (**E**) Kaplan–Meier survival analysis comparing patients with high and low immune scores. Statistical analysis was performed by log-rank test. (**F**) MCPcounter analysis of MYCN-A and MYCN-NA samples showing tumor infiltration of various immune cells. Statistical significance was assessed using the Wilcoxon rank-sum test with Bonferroni correction in (**A**–**C**,**F**). *** *p* < 0.001.

Building on these findings, we further assessed differences in the immune microenvironment using the ESTIMATE (Estimation of STromal and Immune cells in MAlignant Tumor tissues using Expression data) algorithm. MYCN-A samples demonstrated lower immune (Figure 2A), stromal (Figure 2B), and overall ESTIMATE scores (Figure 2C), indicating a reduced immune and stromal cell presence, as well as a lower proportion of non-tumor components within these tumors.

To assess the relationship between immune infiltration and overall survival, samples were stratified into ImmuneScore^High^ and ImmuneScore^Low^ groups. Patients with MYCN-NA tumors predominantly clustered in the ImmuneScore^High^ group (*n* = 366), while MYCN-A tumors were more evenly distributed between the ImmuneScore^High^ (*n* = 47) and ImmuneScore^Low^ (*n* = 45) groups (Figure 2D). Kaplan–Meier survival analysis indicated significantly poorer survival for patients with a low immune score compared to those with a high immune score (Figure 2E). To gain further insight into the specific immune cell populations contributing to these differences, we performed MCPcounter analysis. This analysis revealed that MYCN amplification was strongly associated with the significantly reduced tumor infiltration of T cells, NK cells, myeloid dendritic cells, and monocytic lineage cells (Figure 2F). These results demonstrate that reduced immune cell infiltration in MYCN-amplified tumors contributes to poorer survival outcomes, highlighting the impact of MYCN amplification on the tumor immune microenvironment and patient prognosis.

**Figure 3:**
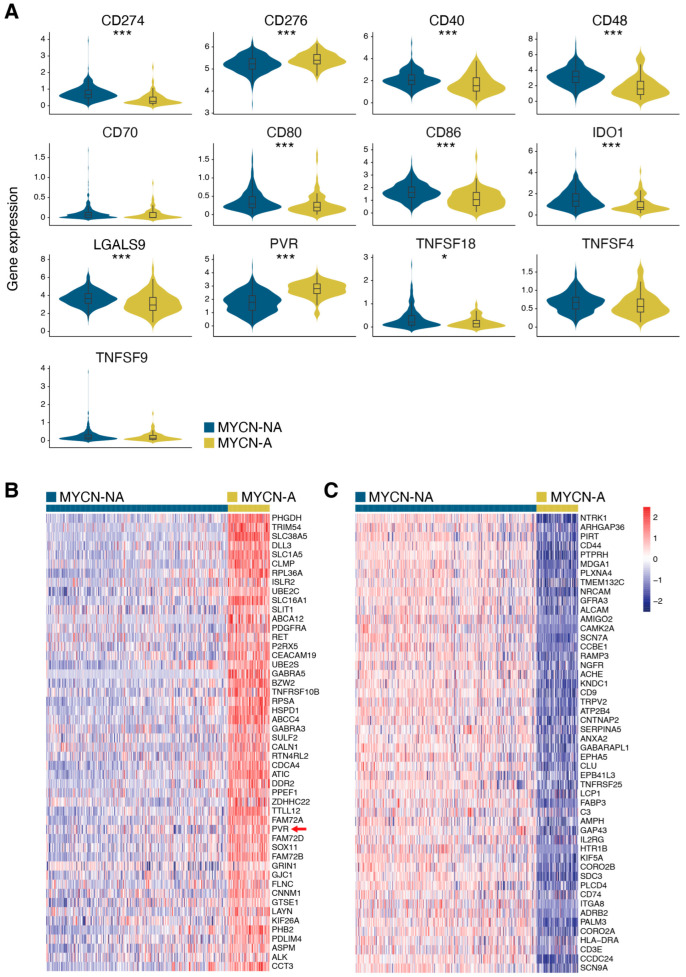
Immune checkpoint and cell surface marker expression in neuroblastoma. (**A**) Gene expression profiles of already described immune check point markers for MYCN-A samples compared to MYCN-NA samples. Statistical significance was assessed using the Wilcoxon rank-sum test with Bonferroni correction. * *p* < 0.05; *** *p* < 0.001. Gene expression heatmaps of the top 50 differentially upregulated (**B**) and downregulated (**C**) cell surface genes, with samples sorted for MYCN status. Already described immune check point markers from A were marked with an arrow.

We next sought to investigate the potential mechanisms by which MYCN-A neuroblastoma tumors may evade immune detection. Therefore, the expression of immune checkpoint-related genes known to modulate immune responses in cancer was assessed. MYCN-A tumors showed significant upregulation of PVR (Nectin-2) and a slight increase in CD276 (B7-H3), both of which inhibit immune cell activity [23,24]. Additionally, MYCN-A tumors exhibited the downregulation of immune-activating genes such as CD48 and CD86, and a slight decrease in CD40 expression, potentially contributing to an immunosuppressive tumor microenvironment. Interestingly, canonical immune checkpoint molecules such as CD274 (PD-L1), which is known to be upregulated in various cancers to promote immune escape [25], was not upregulated in MYCN-A tumors. Notably, the immune-suppressive gene LGALS9, known for its role in dampening immune responses by interacting with TIM-3 on T cells [26], was downregulated (Figure 3A). A further examination of cell surface proteins (Supplementary Table S1) revealed that among the top 50 upregulated cell surface genes, only PVR was found to potentially influence the immune response (Figure 3B). An enrichment analysis of the top 250 upregulated genes highlighted associations with the molecular functions GTPase binding, glycosaminoglycan binding, transmembrane receptor protein tyrosine kinase activity, and carboxylic acid transporter activity—processes that may support tumor growth, metastasis, and metabolic adaptation. Several upregulated ABC transporters, such as ABCA12, ABCC1 and ABCC4, were also identified, which may contribute to immune-modulatory molecule efflux and further alter the tumor microenvironment (Figure 3B and Supplementary Figure S2A). In contrast, the downregulation of HLA-DRA, HLA-A, HLA-B, and HLA-C, which are components of the MHC antigen presentation machinery, suggests a disruption in antigen presentation pathways, further hindering the immune system’s ability to recognize and eliminate tumor cells (Figure 3C and Supplementary Figure S2B). Furthermore, the downregulation of integrins such as ICAM1, which has been shown to be important in anti-cancer immunity [27], was also observed (Supplementary Figure S2B).

**Figure 4:**
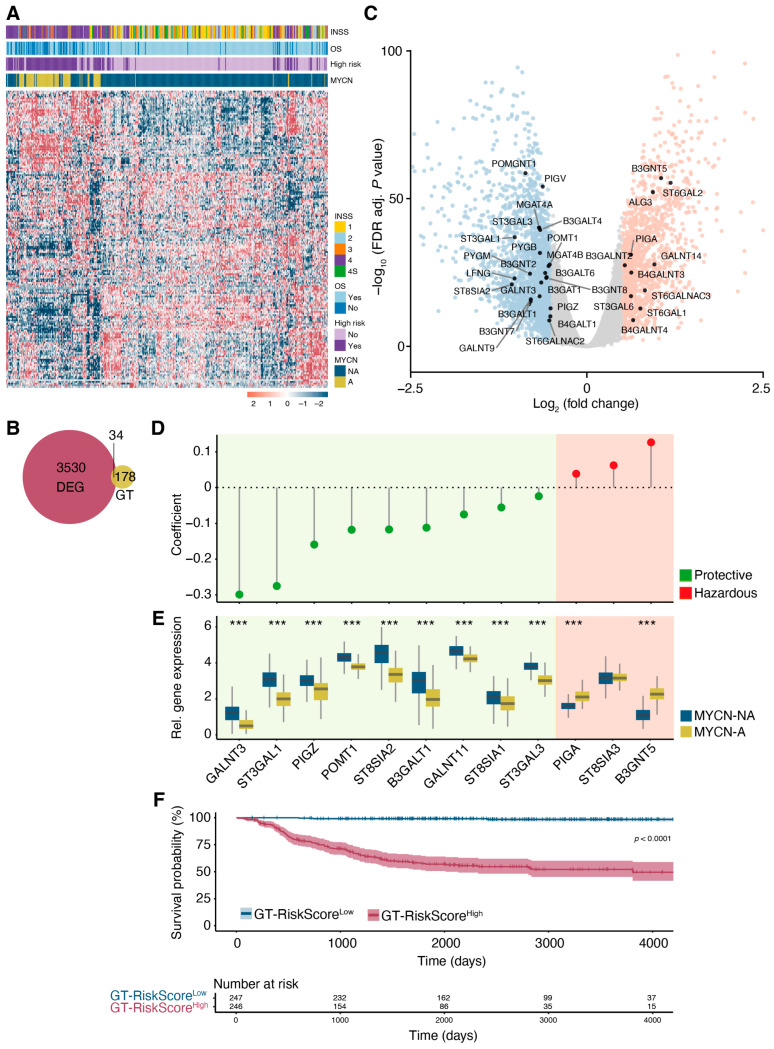
The prognostic risk signature of altered glycosyltransferase expression predicts survival outcomes in neuroblastoma patients. (**A**) Gene expression heatmap with unsupervised hierarchical clustering of glycosylation-associated genes (Gene Ontology Term GO:0070085) across all samples. OS, overall survival. (**B**) Venn diagram representing the overlap between glycosyltransferase genes and DEGs in MYCN-A compared to MYCN-NA samples. (**C**) Volcano plot showing DEGs between MYCN-A and MYCN-NA samples. Genes are categorized as upregulated (logFC > 0.5, adj. *p*-value < 0.01, shown in red), downregulated (logFC < −2.5, adj. *p*-value < 0.01, shown in blue), or not significant (shown in grey). Differentially regulated glycosyltransferase genes are labelled. (**D**) LASSO regression identified 12 glycosyltransferase genes with protective (green) or hazardous (red) effects on survival, as determined by their regression coefficients. (**E**) Boxplots showing the expression of LASSO-selected glycosyltransferase signature genes in MYCN-A and MYCN-NA samples. Statistical significance was assessed using the Wilcoxon rank-sum test with Bonferroni correction. *** *p* < 0.001. (**F**) Kaplan–Meier survival analysis comparing high-risk and low-risk patient groups stratified by risk scores derived from the glycosyltransferase gene signature expression (GT-RiskScore). Statistical significance was determined using the log-rank test.

Given that alterations in cell surface glycosylation can impact key biological processes such as cell–cell recognition and the immune response [16], we aimed to investigate the gene expression patterns associated with glycosylation. Therefore, we examined the expression of genes within the GO glycosylation gene set (GO:0070085). The analysis revealed distinct glycosylation-related gene expression profiles that clearly differentiated MYCN-A samples from MYCN-NA samples (Figure 4A), suggesting a potential link between the MYCN status and altered glycosylation patterns in neuroblastoma. Given that glycosylation is primarily mediated by glycosyltransferase activity, we compared the differential expression of glycosyltransferase genes between MYCN-A and MYCN-NA samples, revealing 34 glycosyltransferase genes with significant differential expression (Figure 4B,C).

To evaluate the prognostic relevance of glycosyltransferase (GT) gene expression in neuroblastoma outcomes, we performed a univariate Cox regression analysis on 212 GT genes. This analysis identified 52 genes with a potential impact on survival. Among these, 13 GT genes were associated with a hazardous effect on survival, while the others were linked to a protective effect, as indicated by their hazard ratios (Supplementary Figure S3A). To refine this prognostic signature and identify independent predictors, a LASSO (Least Absolute Shrinkage and Selection Operator) regression was applied, resulting in a 12-gene GT signature with strong prognostic significance. This signature included nine protective genes (GALNT3, ST3GAL1, PIGZ, POMT1, ST8SIA2, B3GALT1, GALNT11, ST8SIA1, ST3GAL3) and three hazardous genes (PIGA, ST8SIA3, B3GNT5) (Figure 4D). Notably, a comparative expression analysis revealed that all protective genes were downregulated in MYCN-A samples, while hazardous genes were upregulated (Figure 4E).

We then used the expression values of these 12 signature genes, along with their LASSO-derived coefficients, to establish a GT-related risk score (GT-RiskScore). Patients were stratified into GT-high-risk (GT-RiskScore^High^) and GT-low-risk (GTRiskScore^Low^) groups based on their median risk score. Higher risk scores were associated with reduced survival times (Supplementary Figure S3B). The Kaplan–Meier survival analysis demonstrated significantly poorer overall survival for patients in the GT-RiskScore^High^ group compared to those in the GTRiskScore^Low^ group (Figure 4F).

**Figure 5:**
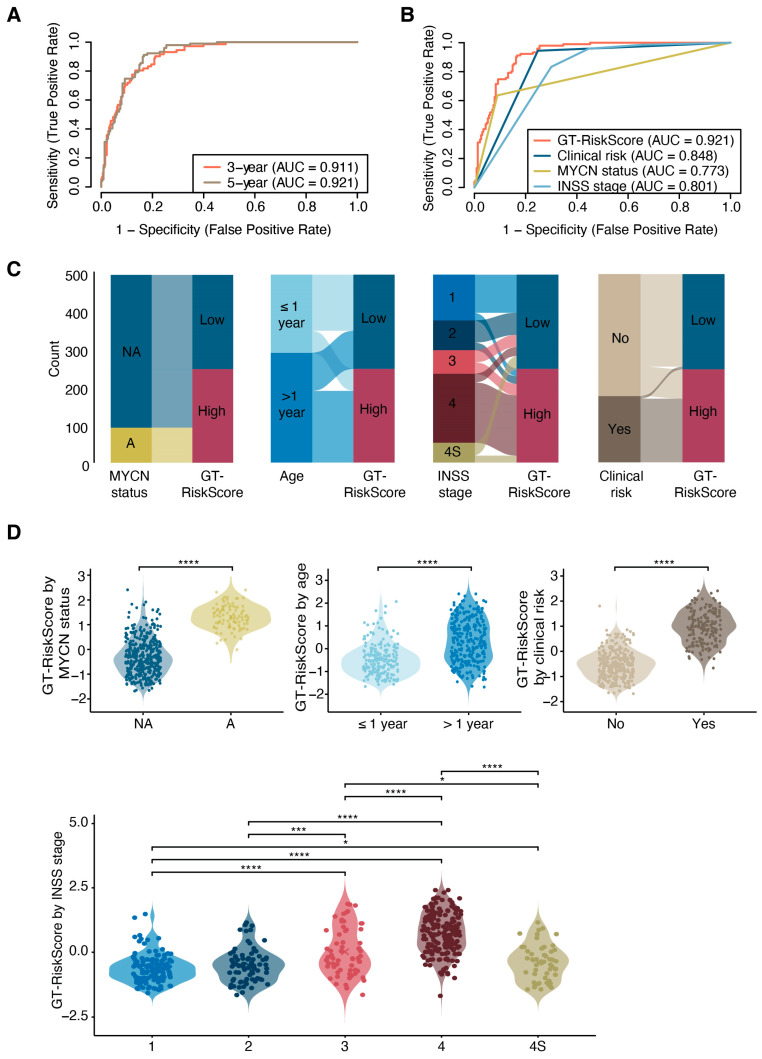
Glycosyltransferase-based risk score (GT-RiskScore) predicts survival outcomes in neuroblastoma patients and correlates with adverse clinical features. (**A**) Time-dependent receiver operating characteristics (ROC) curve analysis for 3-year and 5-year survival predictions based on the glycosyltransferase-based risk score. Corresponding area under the curve (AUC) values are shown. (**B**) ROC curves comparing predictive accuracy of the glycosyltransferase-based risk score, clinical status of high-risk patients, MYCN amplification status and INSS stage for 3-year survival. (**C**) Alluvial plot showing dependency between the GT-RiskScore and different clinical characteristics, including the MYCN amplification status, age at diagnosis, INSS stage and clinical status of high-risk patients. (**D**) Comparison of risk scores between samples based on MYCN status, age at diagnosis, clinical high risk status and INSS stage. NA, non-amplified. A, amplified. Statistical significance was assessed using the Wilcoxon rank-sum test for two-group comparisons. For multiple group comparisons, pairwise differences were evaluated with Bonferroni-corrected Wilcoxon tests. * *p* < 0.05, *** *p* < 0.001, **** *p* < 0.0001.

To evaluate the predictive power of the GT-related risk signature, we performed a time-dependent ROC (Receiver Operating Characteristic) analysis, which revealed AUC (Area Under the Curve) values of 0.911 and 0.921 for 3-year and 5-year overall survival predictions, respectively (Figure 5A). Notably, the GT-related risk signature demonstrated a higher predictive accuracy for 3-year overall survival compared to several established clinical prognostic factors, including the INSS stage, MYCN status, and clinical high-risk categorization (Figure 5B), suggesting its potential use as a valuable prognostic tool in neuroblastoma. Additionally, we analyzed the association of the GT-RiskScore with clinical parameters, including the patient’s MYCN amplification status, age at diagnosis, INSS stage, and clinical high-risk status. Patients with adverse clinical features—such as MYCN amplification, INSS stage 4, or a high clinical risk—predominantly clustered in the GT-RiskScore^High^ group (Figure 5C). The glycosyltransferase-based risk scores were significantly higher in MYCN-A compared to MYCN-NA samples. Similarly, significantly higher risk scores were observed in patients older than 1 year compared to those younger than 1 year, as well as in patients with high clinical risk versus no clinical risk. Furthermore, the GT-RiskScore significantly increased with advancing INSS stage, with patients with INSS stage 3 or 4 exhibiting higher scores compared to stage 1, 2 or 4S (Figure 5D).

Given the observed association between the glycosyltransferase-based risk score and clinical factors, its independent prognostic significance was evaluated through univariate and multivariate Cox regression analyses. The analyses confirmed that the GT-related risk signature is an independent prognostic factor for overall survival, even after adjusting for established clinical variables. The identification of the GT-RiskScore as an independent prognostic marker underscores its potential to enhance risk stratification by providing additional prognostic information beyond traditional clinical parameters (Supplementary Figure S3C).

**Figure 6:**
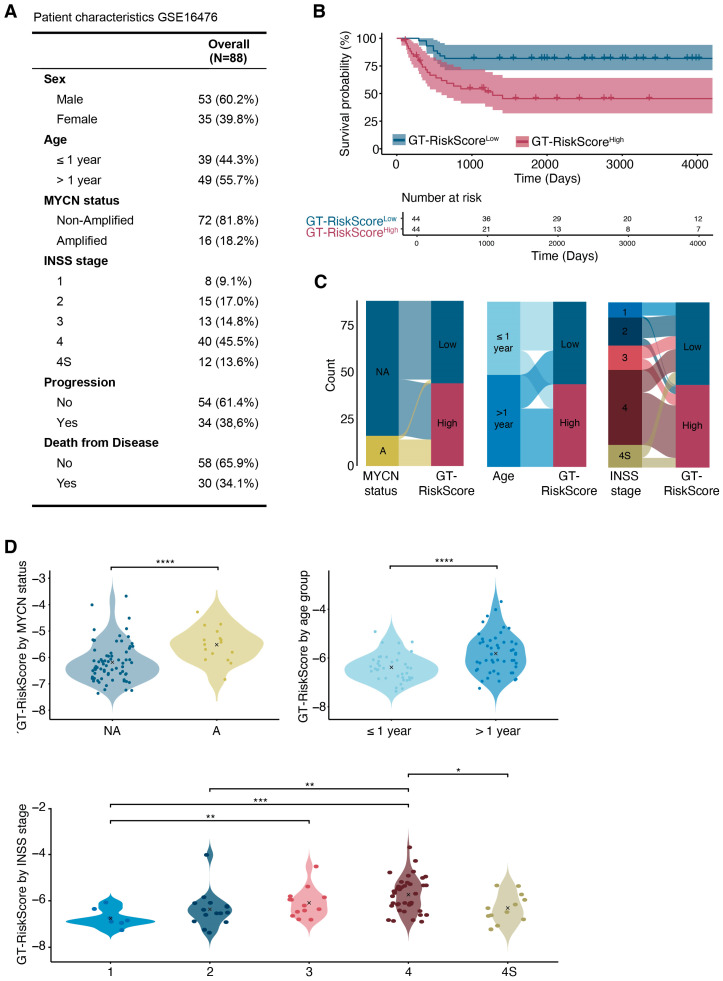
Validation of the glycosyltransferase-based risk score (GT-RiskScore) in the GSE16476 cohort. (**A**) Patient characteristics of the GSE16476 cohort. (**B**) Kaplan–Meier survival analysis comparing high-risk and low-risk patient groups stratified by risk scores derived from the glycosyltransferase gene signature expression (GT-RiskScore). Statistical significance was determined using the log-rank test. (**C**) Alluvial plot showing dependency between the GT-RiskScore and different clinical characteristics, including the MYCN amplification status, age at diagnosis and INSS stage. (**D**) Comparison of risk scores between samples based on the MYCN amplification status, age at diagnosis and INSS stage. NA, non-amplified. A, amplified. Statistical significance was assessed using the Wilcoxon rank-sum test for two-group comparisons. For multiple group comparisons, pairwise differences were evaluated with Bonferroni-corrected Wilcoxon tests. * *p* < 0.05, ** *p* < 0.01, *** *p* < 0.001, **** *p* < 0.0001.

To validate our findings, we evaluated the prognostic significance of the GT-based risk score in an independent neuroblastoma cohort, GSE16476. In this cohort, 18.2% of patients presented with MYCN-A neuroblastoma. The patient distribution by INSS stage was as follows: stage 1 (9.1%), stage 2 (17.0%), stage 3 (14.8%), stage 4 (45.5%), and stage 4S (13.6%). Overall, 65.9% of patients had favorable outcomes, while 34.1% had a poor prognosis (Figure 6A). GT-RiskScores were calculated for all samples and samples were stratified into GT-RiskScore^High^ and GT-RiskScore^Low^ groups based on the median score. Consistent with previous findings in the GSE49711 cohort, Kaplan–Meier survival analysis revealed significantly poorer overall survival for patients in the GT-RiskScore^High^ group compared to those in the GTRiskScore^Low^ group (Figure 6B). We further evaluated the association of the GT-RiskScore and clinical parameters in this cohort. Patients older than 1 year, those with MYCN-A neuroblastoma, and those with INSS stage 4 tumors were predominantly classified into the GT-RiskScore^High^ group (Figure 6C). Additionally, GT-RiskScores were significantly increased in MYCN-A compared to MYCN-NA samples, in patients older than 1 year compared to those younger than 1 year, and in INSS stage 4 tumors compared to those with lower risk stages (Figure 6D). These findings further support the use of GT-RiskScore as a reliable and consistent prognostic marker, demonstrating its predictive value across independent datasets.

## 4. Discussion

Our findings demonstrate the pivotal role of MYCN amplification in neuroblastoma, shaping both gene expression profiles and the immune landscape in ways that directly correlate with clinical outcomes. High-risk neuroblastomas with MYCN amplification show profound immune dysregulation, including altered immune cell infiltration and immune response gene expression, which could suggest a strategy of immune evasion unique to these tumors. Additionally, we identified substantial shifts in the glycosyltransferase gene activity in MYCN-amplified samples, revealing novel glycosylation patterns that may further support tumor immune escape. Together, these findings underscore the dual role of MYCN in both driving aggressive tumor growth and modulating immune susceptibility, highlighting the potential use of glycosylation-related gene signatures as valuable predictive tools in neuroblastoma prognosis.

Our results confirm the well-established association between MYCN, the major key driver of neuroblastoma tumorigenesis, and the poor clinical outcomes in neuroblastoma [12,28]. Patients with MYCN-A tumors exhibited significantly reduced overall survival, while displaying a unique gene expression pattern. Classically, MYCN amplification is associated with the regulation of cell proliferation, differentiation, and apoptosis [10,29]. While its role as a transcription factor already suggests that it has a significant impact on gene expression, our analyses confirmed a massive MYCN-dependent expression change affecting over 3500 genes, indicating its role in regulating the whole transcription machinery [30]. While most of the upregulated genes associated with MYCN amplification fall into well-known categories, the downregulated genes are primarily linked to neuronal processes and, intriguingly, immune responses. The reduction in immune-related gene expression aligns with previous reports indicating that immune evasion plays a key role in the aggressive behavior of MYCN-driven neuroblastoma [31]. The reduced immune infiltration in MYCN-amplified tumors, linked to poor survival outcomes, underscores the role of an immunosuppressive microenvironment in neuroblastoma progression and highlights immune modulation as a potential therapeutic strategy for high-risk patients [10,12,32]. It is therefore of utmost importance to understand the molecular mechanisms underlying neuroblastoma-specific immune evasion to develop targeted therapeutic options. For instance, CAR T cells, which are currently being tested against GD2 in neuroblastoma [33], could be optimized or genetically engineered.

We analyzed known checkpoints and surface markers in MYCN-A versus MYCN-NA tumors to identify factors underlying the reduced immune infiltration in MYCN-A neuroblastomas. While no strongly regulated markers emerged, altered surface proteins, including PVR overexpression, may collectively contribute to immune dysregulation, consistent with PVR’s role as a prognostic marker in other cancers [34]. PVR exerts immunosuppressive effects by interacting with TIGIT, an inhibitory receptor on immune cells [35]. Blocking the PVR-TIGIT axis has demonstrated enhanced anti-tumor immune responses across various cancer types [36], and preclinical data suggest that targeting this pathway could also improve immune responses in neuroblastoma [24]. While there are no current clinical trials that investigate the blocking of the PVR-TIGIT axis in neuroblastoma yet, it could be a promising approach, particularly in combination with the already established anti-GD2 immunotherapy or with GD2-specific CAR T cell strategies [33].

Similarly, we observed the upregulation of CD276 (B7-H3) in MYCN-A neuroblastoma. B7-H3 functions as an inhibitory immune checkpoint molecule, limiting T-cell proliferation and NK cell-mediated cytotoxicity, thereby suppressing immune responses [23,37,38]. B7-H3-specific CAR T cell therapies have shown promising results in xenograft models [39], while dual CAR approaches targeting both B7-H3 and GPC2 have been proposed to address tumor heterogeneity more effectively [40]. The ongoing clinical evaluation of B7-H3-targeted CAR T cell therapies (NCT04691713 and NCT04897321) further supports its relevance as a therapeutic target.

Our study also highlighted the downregulation of immune-activating molecules, such as CD48, which has been linked to decreased NK cell-mediated cytotoxicity, as reported in acute myeloid leukemia [41]. In contrast, we did not observe the upregulation of LGALS9, a molecule involved in regulating T-cell death and proposed as a target for cancer immunotherapy in MYCN-A neuroblastoma [26]. Similarly, PD-L1, a well-known immunosuppressive ligand expressed on cancer cells [25] that activates inhibitory receptors on T cells and NK cells [42], was not upregulated in MYCN-A samples. Nevertheless, anti-PD-1/PD-L1 therapies, while not yet FDA-approved for neuroblastoma (NB), have shown promise in preclinical studies, particularly when combined with agents such as anti-GD2 antibodies, dual checkpoint inhibitors, or CAR T cells, by improving the anti-tumor immune response [43,44,45].

The modulation of cell adhesion and glycosylation further exemplifies how MYCN-A neuroblastoma influences the tumor microenvironment. Altered cell–cell interactions and glycosylation patterns promote immune suppression and metastasis. Specifically, modifications to selectins, siglecs, and integrins can highly impact the ability of tumor cells to interact with other cells and extracellular matrix (ECM) components, thereby facilitating cancer progression [46]. In our study, we observed the downregulation of HLA class I molecules and components of the antigen processing machinery (APM) in MYCN-A tumors, consistent with previous reports of immune evasion through impaired MHC class I expression [47].

Additionally, we detected the upregulation of certain ABC transporters that have not yet been described in the context of immune regulation in neuroblastoma. ABC transporters play a crucial role in modulating the tumor immune microenvironment and contribute to chemoresistance by facilitating drug efflux and cytokine transport [48,49]. In neuroblastoma, ABC transporters are not only linked to drug resistance but also to reduced apoptosis, lower differentiation and increased migration [50].

Integrins also play a critical role in tumor immune escape mechanisms due to their modulation of cell–cell and cell–ECM interactions, which shape the tumor microenvironment [51]. The downregulation of ICAM-1 in MYCN-A neuroblastoma discovered here is particularly noteworthy, given its association with reduced NK cell cytotoxicity and immune escape in other tumors, such as breast cancer [27].

During our analyses, we observed the regulation of numerous genes involved in glycosylation. These genes encode intracellular enzymes that directly influence the composition of the cell surface and can therefore indirectly regulate the immune response. As a result, we examined this class of genes in greater detail. In addition to many MYCN-dependent changes in genes encoding proteins involved in glycosylation, it was particularly intriguing that 34 of the 212 analyzed glycosyltransferases were regulated in a MYCN-dependent manner. The potential relevance of these enzymes for neuroblastoma development becomes particularly evident through our LASSO Cox regression survival prediction model. Using the expression patterns of only 12 genes from the glycosyltransferase family, we were able to develop a prediction model that forecasts the survival probability of neuroblastoma patients with greater accuracy than the INSS stage, risk status, or MYCN status.

Among the glycosyltransferase genes regulated in MYCN-A neuroblastoma, we identified B3GNT5 as a gene with a high hazardous score, significantly upregulated in MYCN-A samples. B3GNT5 catalyzes the addition of N-acetylglucosamine (GlcNAc) to lactosylceramide, forming lactotriaosylceramide, a precursor in the synthesis of lacto and neolacto-series glycosphingolipids [52]. Elevated B3GNT5 activity has been shown to promote the formation of surface neolacto-series glycosphingolipids, which can disrupt HLA class I interactions, potentially impairing CD8^+^ T cell activation and thereby contributing to immune evasion [53]. This immune-modulatory effect, coupled with its pro-tumorigenic roles observed in other cancers such as basal-like breast cancer and pancreatic cancer, underscores the critical impact of B3GNT5 on tumor progression and immune dynamics [54,55].

We also observed the deregulation of PIGA and PIGZ in MYCN-A neuroblastoma. PIG genes are integral to the synthesis of glycosylphosphatidylinositol (GPI) anchors, which tether numerous proteins to the cell membrane, influencing cell signaling, adhesion, and immune interactions [56,57]. The dysregulation of PIGA and PIGZ may alter the function and expression of GPI-anchored proteins, potentially contributing to tumor progression and immune evasion mechanisms. This phenomenon has been highlighted in other cancer contexts, such as hepatocellular carcinoma, where aberrant PIGC expression is linked to poor prognosis through immune modulation and immune cell infiltration [58]. In neuroblastoma, the GPI-anchored oncoprotein GPC2 serves as a therapeutic target, with high expression correlating with poorer survival in high-risk patients [59].

In lung adenocarcinoma, high GALNT3 expression correlates with poorer survival outcomes, but also with elevated immune cell infiltration [60]. In contrast, our findings in MYCN-amplified neuroblastoma show that GALNT3 is downregulated, which coincides with reduced immune cell infiltration. This suggests that GALNT3 may influence immune cell dynamics across various tumor types.

Sialyltransferases, which are key enzymes for synthesizing gangliosides and sialylated glycoproteins, have also been implicated in immune evasion, tumor cell survival, tumor invasion and migration across various tumor types [61]. Among them, ST8SIA3 was identified as differentially regulated in MYCN-A samples, highlighting its role in modifying sialic acids by converting GD3 to GT3 gangliosides [62,63]. In glioblastoma, ST8SIA3 overexpression is linked to the synthesis of the A2B5 sialoganglioside, promoting cancer stem cell properties such as enhanced proliferation and tumor growth [64]. In contrast, ST3GAL1 modulates immune evasion in prostate cancer by synthesizing sialoglycans that interact with Siglec-7 and Siglec-9 receptors on immune cells [65]. In our study, ST3GAL1 downregulation was associated with worse outcomes in neuroblastoma patients.

Interestingly, high ST8SIA1 expression has been correlated with the increased infiltration of CD4^+^ and CD8^+^ T cells, neutrophils, dendritic cells, and M1 macrophages in clear-cell renal cell carcinoma [66]. This is consistent with our observation of reduced ST8SIA1 expression in MYCN-A neuroblastoma, which exhibited lower immune cell infiltration. Moreover, in high-risk and relapsed neuroblastoma, ST8SIA1 suppression by Yes-associated protein (YAP) has been implicated in resistance to GD2-targeted antibody therapies [67]. These findings underscore the complex and prognostic significance of ST8SIA1 in modulating immune responses and therapeutic outcomes.

The aberrant GT gene expression signature detected here aligns with research on other cancers, where alterations in glycosylation pathways have been shown to facilitate immune evasion [61,65]. Changes in glycosylation can disrupt immune recognition by modifying surface molecules, altering antigen presentation, and interfering with immune cell interactions.

While our findings provide a valuable framework for understanding the impact of MYCN amplification on neuroblastoma progression and immune evasion, it is important to acknowledge the inherent limitations of in silico analyses. Computational approaches allow for the identification of broad patterns and relationships across complex datasets, offering unique insights into tumor biology. However, to fully validate the significance of the glycosyltransferase-related signatures and surface markers identified in this study, further research is required. Experimental validation will be critical to elucidate the mechanistic roles of these markers in shaping the tumor microenvironment. The functional roles of glycosyltransferase genes and their impact on tumor–immune interactions and tumor progression can be examined using patient-derived organoids or employing animal models, such as MYCN-driven neuroblastoma mouse models.

Looking ahead, integrating these findings into experimental frameworks will offer a clearer picture of how MYCN drives immune dysregulation and tumor aggressiveness. Such investigations could lead to the development of novel therapeutic strategies targeting glycosylation pathways or optimizing immune-based treatments, such as CAR T cell therapies, to overcome neuroblastoma-specific immune escape mechanisms. Bridging the gap between computational insights and clinical application, our work underscores the importance of a multidisciplinary approach to advance precision medicine for high-risk neuroblastoma patients.

## 5. Conclusions

In conclusion, this study provides compelling evidence that MYCN amplification extends beyond tumor growth to influence immune evasion pathways and glycosylation processes in neuroblastoma, potentially shaping the immune landscape in ways that limit immune engagement. The 12-gene glycosyltransferase risk signature derived here could serve as a robust predictive marker for patient outcomes, offering potential utility in both prognostication and therapeutic targeting. Future studies should aim to further delineate how MYCN-driven glycosylation patterns interact with the immune microenvironment and explore the therapeutic potential of modulating glycosylation pathways in MYCN-amplified neuroblastoma.

## Figures and Tables

**Table 1:** Patient characteristics of neuroblastoma cohort GSE49711.

	Overall (N = 493)
**Sex**	
Male	284 (57.6%)
Female	209 (42.4%)
**Age**	
<18 months	301 (61.1%)
>18 months	192 (38.9%)
**MYCN status**	
Non-Amplified	401 (81.3%)
Amplified	92 (18.7%)
**INSS stage**	
1	120 (24.3%)
2	78 (15.8%)
3	62 (12.6%)
4	181 (36.7%)
4S	52 (10.5%)
**Clinical risk**	
Low	318 (64.5%)
High	175 (35.5%)
**Progression**	
No	313 (63.5%)
Yes	180 (36.5%)
**Death from disease**	
No	389 (78.9%)
Yes	104 (21.1%)

## Data Availability

RNA-seq gene and microarray expression data can be accessed from the GEO database (www.ncbi.nlm.nih.gov/geo/ (accessed on 16 December 2024)) with accession numbers GSE49711 (GSE49711_SEQC_NB_TUC_G_log2) and GSE16476.

## Supplementary Materials

The following supporting information can be downloaded at: https://www.mdpi.com/article/10.3390/jcm14020527/s1, Table S1: Cell surface related genes; Figure S1: MYCN and associated genetic pathway alterations; Figure S2: Gene-concept network plots; Figure S3: Glycosyltransferase genes and risk score.
