# Supplementary material for: Genetic Risk Profiling Reveals Altered Glycosyltransferase Expression as a Predictor for Patient Outcome in Neuroblastoma
Source: J Clin Med. 2025 Jan 15;14(2):527. doi: 10.3390/jcm14020527 (PMC11766279; doi:10.3390/jcm14020527)
Supplement: Supplementary file 1 [file jcm-14-00527-s001.zip › Supplementary Figures.pdf]

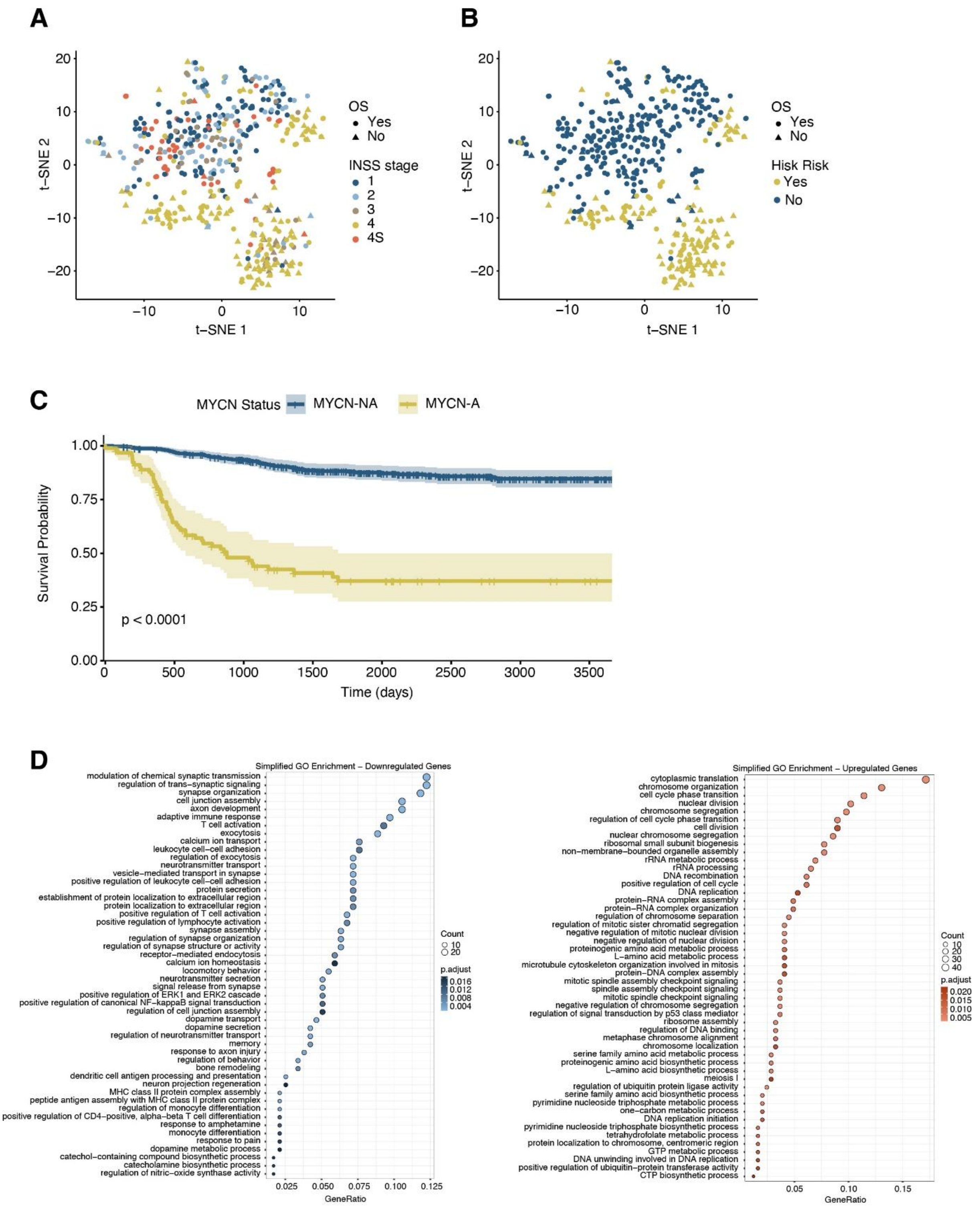

Supplementary Figure S1 | MYCN and associated genetic pathway alterations. **A**, **B**, t-SNE plots of 493 neuroblastoma samples from the GSE49711 cohort showing distribution based on overall survival, INSS stage or high-risk status. **C**, Kaplan-Meier survival analysis comparing MYCN-A and MYCN-NA patients. Statistical significance was assessed using the log-rank test. **D**, Top 50 enriched GO terms for downregulated (left, blue) and upregulated (right, red) DEGs between MYCN-A and MYCN-NA samples.

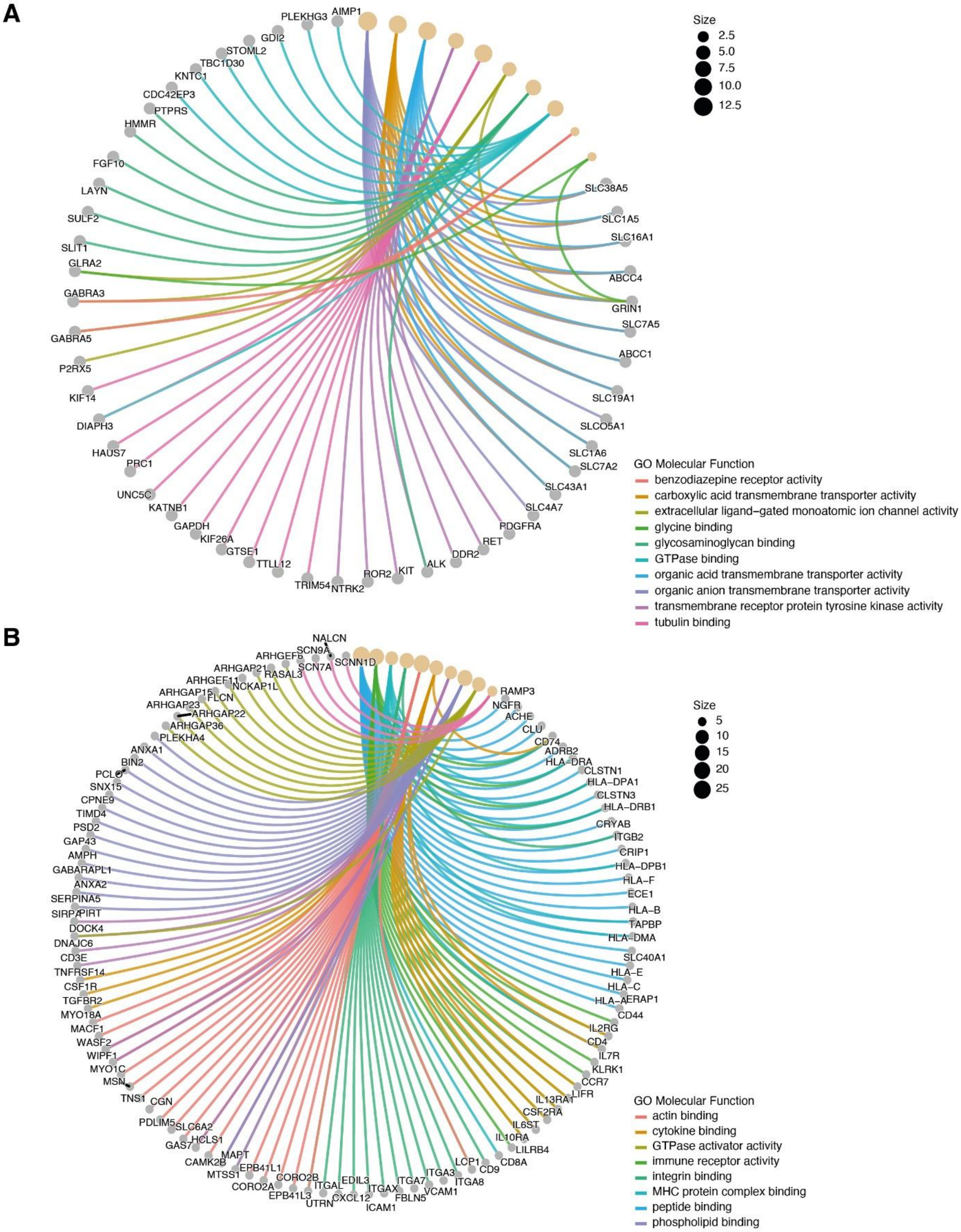

Supplementary Figure S2 | Gene-concept network plots. Illustration of the connections between differentially upregulated (**A**) and downregulated (**B**) cell surface genes and their associated top 10 GO molecular function terms based on adjusted *p*-values. The analysis was performed with the 250 most up- and 250 most downregulated genes.

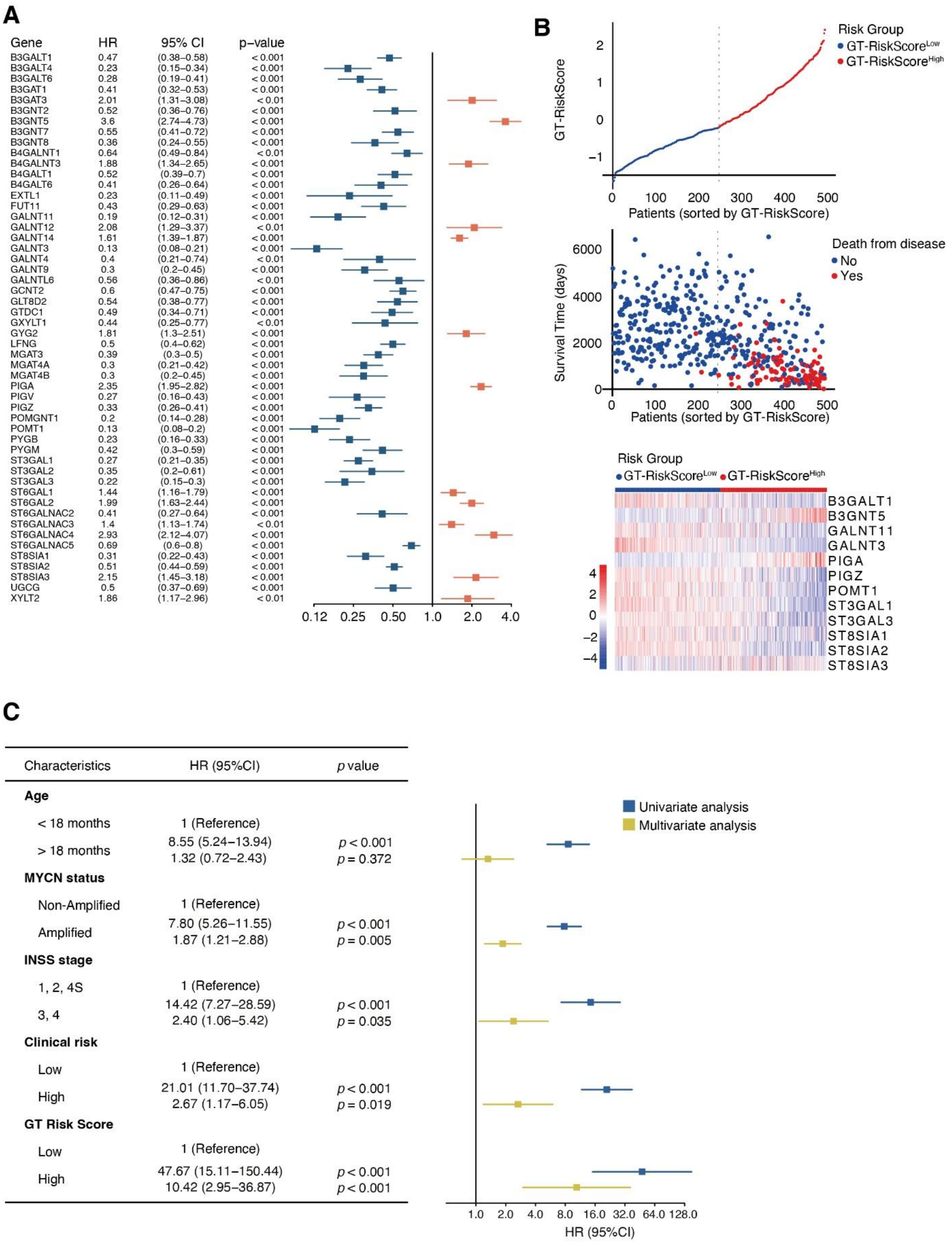

Supplementary Figure S3 | Glycosyltransferase genes and risk score. **A**, Forest plot of hazard ratios (HR) with 95% confidence intervals (CI) for glycosyltransferase genes identified by univariate Cox regression analysis. Genes with protective effects are shown in blue, while hazardous genes are shown in red. **B**, Glycosyltransferase-based risk score (GT-RiskScore) distribution and survival status of patients in high- and low-risk groups, with a heatmap displaying the expression of glycosyltransferase genes associated with the risk profile. **C**, Independent prognostic analysis of the GT-RiskScore using univariate (blue) and multivariate (yellow) Cox regression models, including clinical characteristics.
